# Model Predictive Control of a Novel Wheeled–Legged Planetary Rover for Trajectory Tracking

**DOI:** 10.3390/s22114164

**Published:** 2022-05-30

**Authors:** Jun He, Yanlong Sun, Limin Yang, Feng Gao

**Affiliations:** State Key Laboratory of Mechanical System and Vibration, School of Mechanical Engineering, Shanghai Jiao Tong University, Shanghai 200240, China; sunyanlong@sjtu.edu.cn (Y.S.); ylm20159@sjtu.edu.cn (L.Y.); fengg@sjtu.edu.cn (F.G.)

**Keywords:** mobile robot, advanced intelligent control, wheeled–legged, trajectory tracking, model predictive control

## Abstract

Amid increasing demands for planetary exploration, wide-range autonomous exploration is still a great challenge for existing planetary rovers, which calls for new planetary rovers with novel locomotive mechanisms and corresponding control strategies. This paper proposes a novel wheeled–legged mechanism for the design of planetary rovers. The leg suspension utilizes a rigid–flexible coupling mechanism with a hybrid serial–parallel topology. First, the kinematic model is derived. Then, a control strategy for the wheeled–legged rover that includes a trajectory tracking module based on the model predictive control, the steering strategy, and the wheel speed allocation algorithm is proposed. After that, three groups of cosimulations with different trajectories and speeds, and experiments are carried out. Results of both the simulations and experiments validate the proposed control method.

## 1. Introduction

The planetary rovers that were deployed for the exploration of the moon and Mars, such as Curiosity and Perseverance, are purely wheeled robotic systems [1,2]. They adopt the passive rocker–bogie suspension configuration. There are two identical linkage mechanisms on each side of the rover, which consist of a rocker and a bogie. A differential mechanism is adopted to connect the two linkage mechanisms. One wheel is fixed at one end of the rocker, while the bogie has two wheels that are mounted on the other end of the rocker. Recently, China’s Zhurong Mars rover adopted an active rocker–bogie suspension. There was a novel angle-adjusting mechanism between the two rockers to generate a wheel-step motion that could help the rover avoid wheel slip sinkage [3]. Although many remarkable achievements have been made in the field of planetary exploration, the capability of wide-range autonomous exploration is still a great challenge for planetary rovers.

The hybrid leg–wheel mechanism can be used in the design of planetary rovers. Legged–wheeled robots have the merits of being both wheeled and legged robots. They can robustly deal with uncertainties or disturbances caused by the unstructured discontinuous terrain encountered during planetary exploration. Moreover, they have a relatively high locomotion efficiency. There are three categories of leg–wheel robotic systems that differ according to leg morphology [4]. The first one is the serial leg configuration. For example, the Jet Propulsion Laboratory (JPL) developed an articulated–wheeled lunar robot called ATHLETE [5]. Each leg was a 6R (rotational joint) serial mechanism with six degrees of freedom (DOFs). It could roll over flat smooth terrain on rotating wheels and could also use the wheels as feet to walk over irregular and steep terrain. Grand et al. [6] addressed a wheeled–legged robot called Hylos, which had 16 actively actuated DOFs, with each leg combining a two-DOF leg and the steering and rotation DOFs in the wheel. Smith et al. [7] presented the PAW, a four-legged vehicle with a T-shaped body and compliant legs. Each leg had two DOFs, including a passive prismatic joint. The second one was a leg with a parallel topology. Xu et al. [8] proposed a parallel legged–wheeled robotic system called BIT-NAZA, which had four parallel platforms with six DOFs and a 6-UPU (universal–prismatic–universal joints) configuration. There were four active wheels that were mounted on the feet of the parallel legs. Compared to their serial counterparts, parallel legged–wheeled systems usually have more payload and stiffness [9]. In addition, the actuators of the parallel leg system can be installed on the body, and the inertia of the moving part of the leg can be reduced. The electric devices comprising the actuator, such as the encoder and the torque sensor, can be easily protected [10]. Finally, there is the third category of hybrid wheeled–legged robots, namely transformable wheeled–legged vehicles, such as the Whegs series [11,12], Quattroped [13], Wheel Transformer [14], TurboQuad [15], and STEP [16]. For these robots, the wheel and leg morphology can be switched via the active joints. Transformable leg–wheel robots often adopt simple mechanical structures to simplify the control strategy. Thus, the stability and maneuverability of hybrid robots are inevitably sacrificed [17].

In spite of the excellent kinematic characteristics of the leg mechanisms discussed above, they cannot be directly applied to extraterrestrial exploration rovers because of the existence of special requirements such as maneuverability and security. For example, planetary rovers such as Curiosity [18] have additional wheels and legs to maintain a high level of security to overcome the tough terrains of the outer planet. Exploration rovers must have a fault tolerance feature to ensure the safety of the vehicle. These rovers can continue to move and carry out exploration missions even if one or multiple actuators are not working. For instance, the wheeled–legged rover ShearpTT [19] adopted self-locking gears in the actuator design for the suspension. There are two benefits to this: The first one is that the rover would not fall down when the actuator in the knee joint is invalid. The other is that the rover can support its own weight through the self-locking mechanism without the need for additional electrical energy due to the motor brake.

Legged suspension can change the center of gravity, the body posture, the distribution of contact forces, and even raise wheels to negotiate obstacles. Hence, controlling wheeled–legged robots is more complex than traditional exploration rovers with rocker–bogie suspension, especially in terms of trajectory tracking. Lamon et al. [20] proposed a control method for three-dimensional trajectory tracking. Furthermore, feedback control based on stereo vision efficiently improved the accuracy of trajectory tracking [21,22]. For rovers with independent front and rear steering and four wheels that are driven independently, path tracking becomes more complicated. Krid et al. [23] developed a dynamics-based tracking controller on a horizontal plane using a linear quadratic regulator (LQR). LQR controllers are able to track the line trajectory with quite a good accuracy. However, there is an obvious decrease in the accuracy with regard to steering. In contrast, model predictive control (MPC) can handle complex trajectories [24]. The control algorithm based on MPC can be derived in a recursive form, which is computationally more efficient than the other methods. The computing efficiency is a key evaluation index for planetary rovers because there are very limited computing resources in space. Though the MPC method has been applied to wheeled robots [25,26], there are some differences for wheeled-legged robots. First, wheeled-legged robots possess terrain-adaptive capabilities [27]. The leg length can be adjusted by changing the knee joint angle even if the wheel is always under the hip joint. The attitude angles of the robot can be controlled through changing the leg lengths. Thus, the terrain-adaptive capability needs to be involved in the trajectory tracking when the robot runs across irregular terrains. Second, having wheels that drive independently can lead to an uneven speed distribution, resulting in the occurrence of wheel slip [28]. For wheeled–legged rovers, the pose of the body, the wheel–soil contact force, and the height of the gravitational center can all be adjusted by coordinating the motion of the hip and knee joints when the wheeled–legged robot moves over rough terrain [29]. Both the motion of the hip and knee joints affect the motion characteristics of the wheel, resulting in slippage. Therefore, it is necessary to provide a suitable speed for each wheel that is based on the motion characteristics of the whole robot on rough terrain.

In this paper, a novel wheeled–legged mechanism called TAWL is proposed for the design of planetary rovers. The leg suspension utilizes a rigid–flexible coupling mechanism with a hybrid serial–parallel topology. A kinematic model is derived first. Then, a control strategy for a wheeled–legged rover is proposed that includes a trajectory-tracking module based on MPC, the steering module, and the wheel speed allocation module. After that, a cosimulation model is established in both NX/Motion and Simulink software to verify the control strategy. Finally, experiments are also carried out to validate the proposed control method.

The remainder of the paper is organized as follows: Section 2 reports the hardware design and the kinematics of the rover; Section 3 details the control strategy; Section 4 presents the simulations, experiments, and the discussion of the results; and finally, Section 5 offers the conclusions.

## 2. Hardware and Kinematics of the Rover

### 2.1. Mechanical Structure

**Figure 1:**
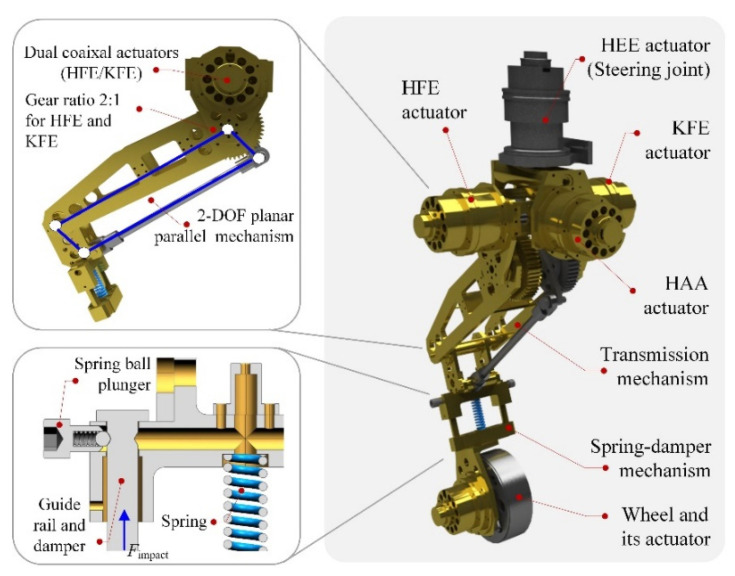
The leg–wheel structure.

There are two aspects that need to be considered for leg design: First, the leg inertia must be as low as possible. Each leg has four DOFs, as illustrated in Figure 1, namely the hip abduction/adduction (HAA) joint, the hip flexion/extension (HFE) joint, the hip endo/exorotation (HEE), and the knee flexion/extension (KFE) joint. The HEE joint can also be used to steer the wheels. To reduce the rotational inertia of the robot’s legs, the actuators of the HFE and KFE joints are coaxially located at the hip. The KFE joint is actuated by a pantograph mechanism. In addition, to increase the driving torques of the HFE and KFE joints, a gear reducer stage was adopted at each of their output shafts. Second, compliant mechanisms are necessary for legged robotic systems to handle uncertainties or disturbances such as ground contact collisions. A telescopic structure with a passive damped spring was used for the leg design. In addition, there are two spring ball plungers that trigger the spring–damper mechanism. When the impact force from the ground exceeds the threshold value of the spring ball plunger, the spring–damper mechanism works to dissipate the impact energy. After that, the lower leg returns to its original length with the restoring force of the spring.

**Figure 2:**
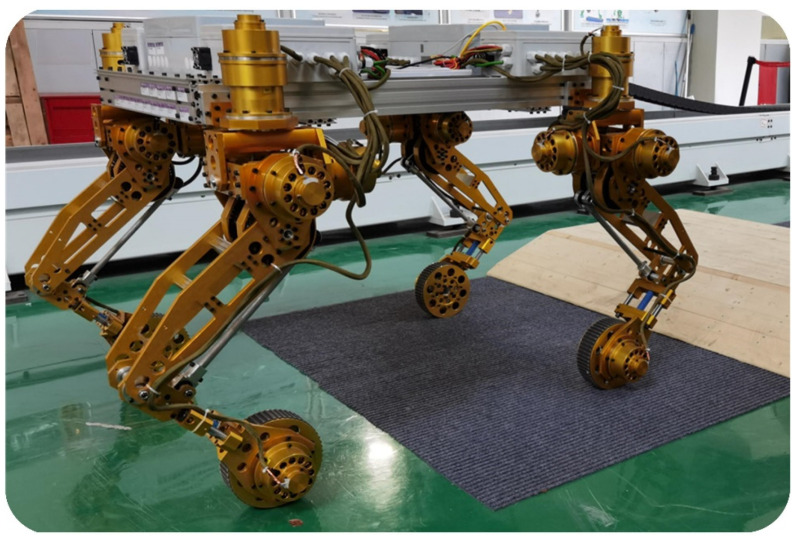
The TAWL robot.

The TAWL robot has four identical legs with wheels. The four legs are mounted to the torso in an axially symmetric distribution, as depicted in Figure 2. Its four hip joints are located on a circle with a diameter of 1.2 m. From a biological view, to obtain highly dynamic characteristics in the longitudinal direction, the ratio of the length to the width of the torso should be more than 1. However, the wheeled mode is the primary motion mode for the TAWL robot. An axially symmetrical arrangement was adopted for the robot design so that the robot would have all-directional locomotion capability in both the wheeled and legged modes (walking or trotting). Furthermore, this arrangement also increases the number of legged locomotion modes. There are at least three leg configurations for legged locomotion, i.e., the M-configuration, O-configuration, and X-configuration.

### 2.2. Perception and Control System

**Figure 3:**
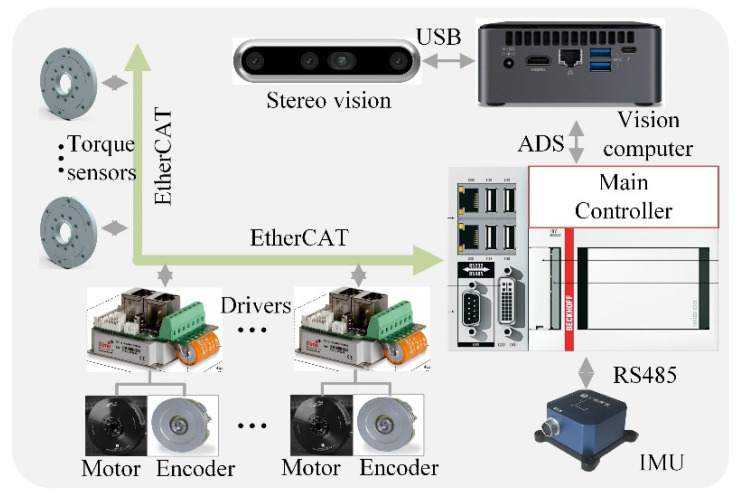
The perception and control system.

There are two types of sensors: proprioceptive and exteroceptive sensors. Proprioceptive sensors contain the joint encoder, the joint torque sensor, and the inertial measurement unit (IMU), as seen in Figure 3. All of the joint angles are precisely measured by absolute encoders. Since the angle measures of each motor are absolute, the robot does not have to be homed at startup. The IMU sensor is mounted on the body and is responsible for the poses of the robot’s torso. Exteroceptive sensors include visual and nonvisual sensors, which are employed to measure environmental information such as the geometrical parameters of the terrain and ground contact forces. Here, a stereo vision system was attached to the front part of the main body. Furthermore, an independent computer was implemented to deal with the vision algorithms. To improve the reliability, we did not assemble force sensors for ground contact force measurements to the end of each leg. We established a distribution measurement model and then evaluated the ground contact forces using the measurement data from the joint torque sensors.

An onboard main controller was used to run the entire control program. The main controller communicates with 20 servo drives and 16 joint torque sensors in real time via the EtherCAT industrial network protocol (Bechoff, Verl, Germany). The measurement data from the IMU sensor are transferred into the main controller according to the RS-485 serial data standard. The main controller communicates with the visual controller by means of the ADS (automation device specifications) protocol. The TAWL robot’s control software was developed using the TwinCAT software platform (Bechoff, Verl, Germany), a real-time PC-based control system. In addition, there are two on-board lithium batteries that the robot can use to run for about 1.5 h.

### 2.3. Kinemactics of the Rover

**Figure 4:**
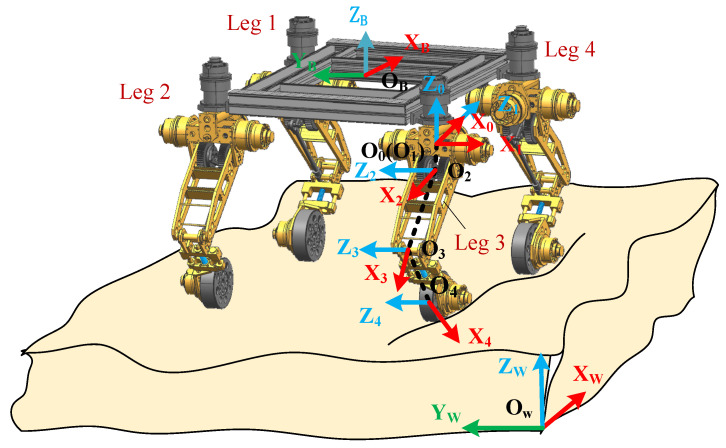
The coordinate systems of the rover.

The body frame {O_B_—X_B_Y_B_Z_B_} of the whole robot is located at the center of the plane and is composed of the centers of four hip joints. We established the D-H coordinate systems in Figure 4 for each leg. Because each leg has the same kinematic structure, the D-H parameters of the four legs are also the same. {O_0_—x_0_y_0_z_0_} is the base frame of each leg (i.e., the leg frame), which is located at the center of the hip joint. {O_4_—x_4_y_4_z_4_} is the wheel frame of each leg. The D-H parameters are shown in Table 1. Therefore, the transformation matrix from frame *i* − 1 to *i* for the *i*th limb can be written as

(1) Tii−1=[cθi−sθicαisθisαiaicθisθicθicαi−cθisαiaisθi0sαicαidi0001]

where *s* and *c* denote the sine and cosine functions.

For the *i*th leg, the transformation matrix from the wheel frame to the leg frame is written as

(2) T40i=T10iT21iT32iT43i=(RP01)=(−s34−c340−L3s34−L2s3c34c2−s34c2−s2c2(L1+L3c34+L2c3)c34s2−s34s2c2s2(L1+L3c34+L2c3)0001),

where *s*_34_ = sin(*θ*_3_+*θ*_4_), *c*_34_ = cos(*θ*_3_+*θ*_4_), *s*_2_ = sin*θ*_2_, *c*_2_ = cos*θ*_2_, *s*_3_ = sin*θ*_3_, and *c*_3_ = cos*θ*_3_. Here, *θ*_1_ = π/2 and $P={\left(P_{x},\;P_{y}\;P_{z}\right)}^{T}$. are the positions of the wheel center with respect to the leg frame.

When ***P*** is given, the rotational angle of each joint, *θ*_2_, *θ*_3_, and *θ*_4_, can be obtained as

(3) $$\theta_{2}=\mathrm{atan}(P_{z}/P_{y}),$$

(4) $$\theta_{3}=-\mathrm{atan}\left(\frac{C}{A^{2}+B^{2}+C^{2}}\right)-atan\left(\frac{B}{A}\right),$$

(5) $$\theta_{4}=-\mathrm{atan}\left(\frac{G}{\sqrt{E^{2}+F^{2}-G^{2}}}\right)-atan\left(\frac{F}{E}\right)-\theta_{3}$$

where

$$A=2P_{x}L_{2}$$

$$B=-2\left(\sqrt{P_{y}{}^{2}+P_{z}{}^{2}}-L_{1}\right)L_{2}$$

$$C={\left(\sqrt{P_{y}{}^{2}+P_{z}{}^{2}}-L_{1}\right)}^{2}+L_{2}{}^{2}+P_{x}{}^{2}-L_{3}{}^{2}$$

$$E=2P_{x}L_{3}$$

$$F=-2L_{3}\left(\sqrt{P_{y}{}^{2}+P_{z}{}^{2}}-L_{1}\right)$$

$$G={\left(\sqrt{P_{y}{}^{2}+P_{z}{}^{2}}-L_{1}\right)}^{2}+P_{x}{}^{2}+L_{3}{}^{2}-L_{2}{}^{2}$$

## 3. Control Strategy

**Figure 5:**
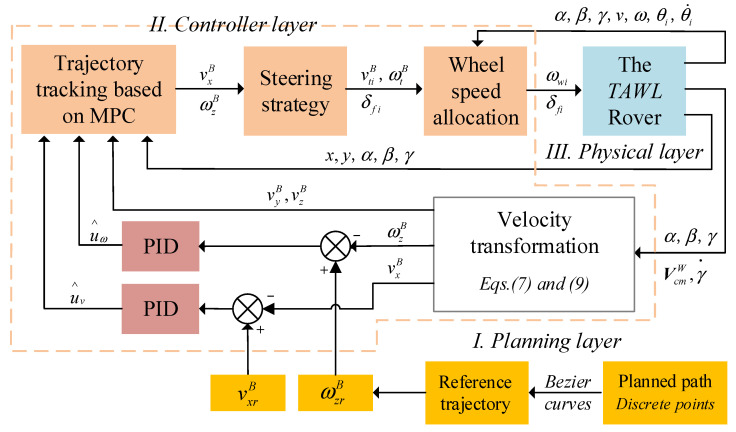
The rover’s control strategy.

In this section, a control architecture for the wheeled–legged rover is proposed, as depicted in Figure 5. The control strategy consists of a planning layer, a controller layer, and a physical layer. First, the planner layer generates the reference trajectory. A planned path is generally composed of discrete points that come from the operator or the planner, which is based on a vision system. Using these points, a Bezier curve was adopted to produce a reference trajectory that included the time information. Thus, the derivation of the reference trajectory yielded the reference velocity. Second, the controller layer includes an MPC module, a steering module, and a wheel speed allocation module. The MPC module calculates the optimal control inputs through the last control inputs and the current state variables. The state variables can be estimated by a data fusion algorithm such as a Kalman filter and a particle filter, which is based on the proprioceptive and exteroceptive sensors in the robotic system. Considering that the main purpose was to verify the trajectory tracking algorithm, the state variables, including the position and velocity of the robot, were measured by the vision motion capture system directly in the present experimental study. In addition, to eliminate the accumulated errors, a PID control method was added in the loop. Then, the steering module provided the speed and the steering angle of each wheel. After that, the wheel speed allocation algorithm was presented to avoid a wheel slip. Next, the leg joint angles were obtained through the inverse kinematics of the rover. Third, the physical layer received the steering angles, the wheel speeds, and the leg joint angles and sent these orders to servo drives.

### 3.1. Locomotive Equations

The rotation matrix from the body frame {B} to the world frame {W} is written as

(6) RBW(α,β,γ)=(cγcβcγsβsα−sγcαcγsβcα+sγsαsγcβsγsβsα+cγcαsγsβcα−cγsα−sβcβsαcβcα),

where α,β, and γ are the fixed rotational angles with respect to the *x*, *y*, and *z* axes of the world frame, respectively.

Furthermore, the velocity of the centroid of the robot can be denoted by

(7) $$V_{cm}^{W}=\left(\begin{array}{c} \dot{x}\\ \dot{y}\\ \dot{z} \end{array}\right)=\left(\begin{array}{ccc} c\gamma c\beta & c\gamma s\beta s\alpha -s\gamma c\alpha & c\gamma s\beta c\alpha +s\gamma s\alpha \\ s\gamma c\beta & s\gamma s\beta s\alpha +c\gamma c\alpha & s\gamma s\beta c\alpha -c\gamma s\alpha \\ -s\beta & c\beta s\alpha & c\beta c\alpha \end{array}\right)V_{cm}^{B},$$

where $V_{cm}^{W}$ and $V_{cm}^{B}$ are the velocities of the body centroid for frames {W} and {B}, and $V_{cm}^{B}={\left(v_{x}^{B},v_{y}^{B},v_{z}^{B}\right)}^{T}$.

The yaw angle of the robot with respect to the body frame, $\gamma^{B}$, can be written as

(8) $$\tan\gamma =\tan\gamma^{B}c\alpha /c\beta$$

where γ is the yaw angle in frame {W}.

Thus, we have

(9) $$\dot{\gamma }=\omega_{z}^{B}c\alpha /c\beta .$$

Aordingly, the kinematic equation for the trajectory tracking is obtained as

(10) $$\dot{X}=\left(\begin{array}{c} \dot{x}\\ \dot{y}\\ \dot{\gamma } \end{array}\right)=\left(\begin{array}{cc} c\gamma c\beta & 0\\ s\gamma c\beta & 0\\ 0 & \frac{c\alpha }{c\beta } \end{array}\right)\left(\begin{array}{c} v_{x}^{B}\\ \omega_{z}^{B} \end{array}\right)+\left(\begin{array}{cc} c\gamma s\beta s\alpha -s\gamma c\alpha & c\gamma s\beta c\alpha +s\gamma s\alpha \\ s\gamma s\beta s\alpha +c\gamma c\alpha & s\gamma s\beta c\alpha -c\gamma s\alpha \\ 0 & 0 \end{array}\right)\left(\begin{array}{c} v_{y}^{B}\\ v_{z}^{B} \end{array}\right)$$

where $\;X={\left(x,y,\gamma \right)}^{T}$, $u={\left(v_{x}^{B},\;\omega_{z}^{B}\right)}^{T}$, and $\dot{X}={\left(\dot{x},\dot{y},\dot{\gamma }\right)}^{T}=f\left(X,u,t\right)$. At a reference point on the trajectory, we have $X_{r}={\left(x_{r},\;y_{r},\;\gamma_{r}\right)}^{T}$, ${\dot{X}}_{r}=f\left(X_{r},\;u_{r},\;t\right)={\left({\dot{x}}_{r},{\dot{y}}_{r},{\dot{\gamma }}_{r}\right)}^{T}$, and $u_{r}={\left(v_{xr}^{B},\;\omega_{zr}^{B}\right)}^{T}$. 

Therefore, expanding Equation (10) in the Taylor series around the reference point ($X_{r},u_{r}$) and discarding the high order terms yields

(11) $$\dot{X}=f\left(X_{r},\;u_{r},\;t\right)+\frac{\partial f\left(X,u,t\right)}{\partial X}{|}_{\left(X_{r}\;,\;u_{r}\right)}\left(X-X_{r}\right)+\frac{\partial f\left(X,u,t\right)}{\partial u}{|}_{\left(X_{r}\;,\;u_{r}\right)}\left(u-u_{r}\right)$$

where

$$\frac{\partial f\left(X,u,t\right)}{\partial X}{|}_{\left(X_{r}\;,\;u_{r}\right)}=\left(\begin{array}{ccc} \frac{\partial f_{1}\left(X,u,t\right)}{\partial x} & \frac{\partial f_{1}\left(X,u,t\right)}{\partial y} & \frac{\partial f_{1}\left(X,u,t\right)}{\partial \gamma }\\ \frac{\partial f_{2}\left(X,u,t\right)}{\partial x} & \frac{\partial f_{2}\left(X,u,t\right)}{\partial y} & \frac{\partial f_{2}\left(X,u,t\right)}{\partial \gamma }\\ \frac{\partial f_{3}\left(X,u,t\right)}{\partial x} & \frac{\partial f_{3}\left(X,u,t\right)}{\partial y} & \frac{\partial f_{3}\left(X,u,t\right)}{\partial \gamma } \end{array}\right)$$

$$=\left(\begin{array}{ccc} 0 & 0 & -s\gamma_{r}c\beta v_{xr}^{B}+\left(-s\gamma_{r}s\alpha s\beta -c\gamma_{r}c\alpha \right)v_{y}^{B}+\left(-s\gamma_{r}s\beta c\alpha +c\gamma_{r}s\alpha \right)v_{z}^{B}\\ 0 & 0 & c\gamma_{r}c\beta v_{xr}^{B}+\left(c\gamma_{r}s\alpha s\beta -s\gamma_{r}c\alpha \right)v_{y}^{B}+\left(c\gamma_{r}s\beta c\alpha +s\gamma_{r}s\alpha \right)v_{z}^{B}\\ 0 & 0 & 0 \end{array}\right),$$

$$\frac{\partial f\left(X,u,t\right)}{\partial u}{|}_{\left(X_{r}\;,\;u_{r}\right)}=\left(\begin{array}{cc} \frac{\partial f_{1}\left(X,u,t\right)}{\partial v_{x}^{B}} & \frac{\partial f_{1}\left(X,u,t\right)}{\partial \omega_{z}^{B}}\\ \frac{\partial f_{2}\left(X,u,t\right)}{\partial v_{x}^{B}} & \frac{\partial f_{2}\left(X,u,t\right)}{\partial \omega_{z}^{B}}\\ \frac{\partial f_{3}\left(X,u,t\right)}{\partial v_{x}^{B}} & \frac{\partial f_{3}\left(X,u,t\right)}{\partial \omega_{z}^{B}} \end{array}\right)=\left(\begin{array}{cc} c\beta c\gamma_{r} & 0\\ c\beta s\gamma_{r} & 0\\ 0 & c\alpha /c\beta \end{array}\right).$$

Accordingly, the state space equation can be denoted by

(12) $$\dot{\hat{X}}=A\left(t\right)\hat{X}+B\left(t\right)\hat{u}$$

where $\hat{X}={\left(x-x_{r},y-y_{r},\gamma -\gamma_{r}\right)}^{T}$, $\dot{\hat{X}}={\left(\dot{x}-{\dot{x}}_{r},\dot{y}-{\dot{y}}_{r},\dot{\gamma }-{\dot{\gamma }}_{r}\right)}^{T}$, $\hat{u}=\left({\hat{u}}_{v},{\hat{u}}_{\omega }\right)={\left(v-v_{xr}^{B},\omega -\omega_{zr}^{B}\right)}^{T}$, $A\left(t\right)=\frac{\partial f\left(X,u,t\right)}{\partial X}{|}_{\left(X_{r}\;,\;u_{r}\right)}$, and $B\left(t\right)=\frac{\partial f\left(X,u,t\right)}{\partial u}{|}_{\left(X_{r}\;,\;u_{r}\right)}$. $\hat{X}$ is the error with respect to the reference trajectory, and $\hat{u}$ is its associated perturbation control input.

Using forward differences, the approximation of $\dot{X}$ can be obtained as the following discrete-time form:

(13) $$\hat{X}\left(k+1\right)=G_{k}\hat{X}\left(k\right)+H_{k}\hat{u}\left(k\right)$$

where $G_{k}=TA_{k}+I$, and $H_{k}=TB_{k}$. *T* and *k* are the sampling period and the sampling time. ***I*** is the identity matrix.

### 3.2. Trajectory Tracking Model Based on MPC

#### 3.2.1. Objective Function

A controller was designed for the wheeled–legged robot to track the desired trajectory precisely and stably. By changing the current and future inputs of the control system, the optimization problem is the minimization of a predicted performance cost, which is a quadratic function of the states and control inputs as follows:

(14) $$J\left(t\right)=\overset{N_{p}}{\underset{i=1}{\sum }}{\hat{X}}^{T}\left(t+i|t\right)Q\hat{X}\left(t+i|t\right)+\overset{N_{c}-1}{\underset{j=1}{\sum }}\Delta {\hat{u}}^{T}\left(t+i|t\right)R\Delta \hat{u}\left(t+i|t\right)+\rho \varepsilon^{2},$$

where *N_p_* and *N_c_* are the prediction and control horizons, respectively. Here, ***Q*** and ***R*** are the weighting matrices; ρ is the weight coefficient, and ε is the relaxation factor.

Let

(15) $$\xi \left(k|t\right)=\left(\begin{array}{c} \hat{X}\left(k|t\right)\\ \hat{u}\left(k-1|t\right) \end{array}\right),$$

we obtain

(16) $$\xi \left(k+1|t\right)={\hat{A}}_{k}\xi \left(k|t\right)+{\hat{B}}_{k}\Delta \hat{u}\left(k|t\right),$$

(17) $$\eta \left(k|t\right)={\hat{C}}_{k}\xi \left(k|t\right),$$

where ${\hat{A}}_{k}=\left(\begin{array}{cc} G_{k} & H_{k}\\ 0 & I \end{array}\right)$, and ${\hat{B}}_{k}=\left(\begin{array}{c} H_{k}\\ I \end{array}\right)$.

Furthermore, Equations (16) and (17) can be rewritten as the following matrix form:

(18) Y(t)=Ωξ(t)+ΦΔU

where

$$Y\left(t\right)=\left(\begin{array}{c} \eta (k+1|t)\\ \eta (k+2|t)\\ \begin{array}{c} \cdots \\ \eta (k+N_{c}|t)\\ \begin{array}{c} \cdots \\ \eta (k+N_{p}|t) \end{array} \end{array} \end{array}\right),\;\Omega =\left(\begin{array}{c} {\hat{C}}_{k}{\hat{A}}_{k}\\ {\hat{C}}_{k}{\hat{A}}_{k}^{2}\\ \begin{array}{c} \cdots \\ {\hat{C}}_{k}{\hat{A}}_{k}^{N_{c}}\\ \begin{array}{c} \cdots \\ {\hat{C}}_{k}{\hat{A}}_{k}^{N_{p}} \end{array} \end{array} \end{array}\right),\;\Delta U=\left(\begin{array}{c} \Delta u(t|t)\\ \Delta u(t+1|t)\\ \begin{array}{c} \cdots \\ \Delta u(t+N_{c}|t) \end{array} \end{array}\right),\;\mathrm{and}$$

$$\Phi =(\begin{array}{cccc} {\hat{C}}_{k}{\hat{B}}_{k} & 0 & 0 & 0\\ {\hat{C}}_{k}{\hat{A}}_{k}{\hat{B}}_{k} & {\hat{C}}_{k}{\hat{B}}_{k} & 0 & 0\\ \cdots & \cdots & \ddots & \cdots \\ {\hat{C}}_{k}{\hat{A}}_{k}^{N_{c}-1}{\hat{B}}_{k} & {\hat{C}}_{k}{\hat{A}}_{k}^{N_{c}-2}{\hat{B}}_{k} & \cdots & {\hat{C}}_{k}{\hat{B}}_{k}\\ {\hat{C}}_{k}{\hat{A}}_{k}^{N_{c}}{\hat{B}}_{k} & {\hat{C}}_{k}{\hat{A}}_{k}^{N_{c}-1}{\hat{B}}_{k} & \cdots & {\hat{C}}_{k}{\hat{A}}_{k}{\hat{B}}_{k}\\ \vdots & \vdots & \ddots & \vdots \\ {\hat{C}}_{k}{\hat{A}}_{k}^{N_{p}-1}{\hat{B}}_{k} & {\hat{C}}_{k}{\hat{A}}_{k}^{N_{p}-2}{\hat{B}}_{k} & \cdots & {\hat{C}}_{k}{\hat{A}}_{k}^{N_{p}-N_{c}-1}{\hat{B}}_{k} \end{array})$$

Equations (14) and (18) yield

(19) $$\begin{array}{c} J\left(t\right)=\Delta U^{T}\left(t\right)R\Delta U\left(t\right)+Y^{T}\left(t\right)QY\left(t\right)+\rho \varepsilon^{2}\\ =\Delta U^{T}\left(t\right)R\Delta U\left(t\right)+{\left(\Phi \Delta U\left(t\right)\right)}^{T}Q\left(\Phi \Delta U\left(t\right)\right)\\ +2{\left(\Omega \xi \left(t\right)\right)}^{T}Q\left(\Phi \Delta U\left(t\right)\right)+{\left(\Omega \xi \left(t\right)\right)}^{T}Q\left(\Omega \xi \left(t\right)\right)+\rho \varepsilon^{2}. \end{array}$$

Here, Ωξ(t) is not affected by the inputs and can thus be discarded. Therefore, the objective function is rewritten as a standard quadratic form:

(20) $$J\left(t\right)=\left(\begin{array}{cc} \Delta U^{T}\left(t\right) & \varepsilon \end{array}\right)H\left(t\right){\left(\begin{array}{cc} \Delta U^{T}\left(t\right) & \varepsilon \end{array}\right)}^{T}+F\left(t\right){\left(\begin{array}{cc} \Delta U^{T}\left(t\right) & \varepsilon \end{array}\right)}^{T},$$

where

$$H\left(t\right)=\left(\begin{array}{cc} \Phi^{T}Q\Phi +R & 0\\ 0 & \rho \end{array}\right),\;F\left(t\right)=\left(\begin{array}{cc} 2{\left(\Omega \xi \left(t\right)\right)}^{T}Q\Phi & 0 \end{array}\right).$$

#### 3.2.2. Constraints

There are some constraints when the wheeled–legged robot carries out trajectory tracking tasks. The amplitude of the control input ***u*** and control input increment Δ***u*** satisfy

(21) $$u_{min}\left(t+k|t\right)\leq u\left(t+k|t\right)\leq u_{max}\left(t+k|t\right),\;k=0,1\cdots N_{c}-1,$$

(22) $$\Delta u_{min}\left(t+k|t\right)\leq \Delta u\left(t+k|t\right)\leq \Delta u_{max}\left(t+k|t\right),\;k=0,1\cdots N_{c}-1,$$

where $u_{min}$ and $\Delta u_{min}$ are the predefined lower bounds, and $u_{max}$ and $\Delta u_{max}$ are the predefined upper bounds. Furthermore, the variable to be solved in the objective function are the control increment in the control horizon. Therefore, the constraints need be converted into the product form of the control increment and the transformation matrix. 

The following relationship exists:

(23) u(t+k|t)=u(t+k−1|t)+Δu(t+k|t).

Furthermore, Equation (23) can be reformulated as a matrix form:

(24) U(t)=EΔU(t)+U(t−1)

where

$$U\left(t\right)=\left(\begin{array}{c} u\left(t|t\right)\\ u\left(t+1|t\right)\\ \vdots \\ u\left(t+N_{c}-1|t\right) \end{array}\right),E=\left(\begin{array}{cccc} I & 0 & 0 & 0\\ I & I & 0 & 0\\ \vdots & \vdots & \ddots & \vdots \\ I & I & I & I \end{array}\right),$$

$$\Delta U\left(t\right)=\left(\begin{array}{c} \Delta u\left(t|t\right)\\ \Delta u\left(t+1|t\right)\\ \vdots \\ \Delta u\left(t+N_{c}-1|t\right) \end{array}\right),U\left(t-1\right)=\left(\begin{array}{c} u\left(t-1\right)\\ u\left(t-1\right)\\ \vdots \\ u\left(t-1\right) \end{array}\right).$$

Moreover, from Equations (19) and (23), we obtain

(25) $$U_{min}\left(t\right)\leq E\Delta U\left(t\right)+U\left(t-1\right)\leq U_{max}\left(t\right),$$

where

$$U_{min}\left(t\right)=\left(\begin{array}{c} u_{min}\left(t|t\right)\\ u_{min}\left(t+1|t\right)\\ \vdots \\ u_{min}\left(t+N_{c}-1|t\right) \end{array}\right),\;U_{max}\left(t\right)=\left(\begin{array}{c} u_{max}\left(t|t\right)\\ u_{max}\left(t+1|t\right)\\ \vdots \\ u_{max}\left(t+N_{c}-1|t\right) \end{array}\right).$$

For the control increment, we have

(26) $$\Delta U_{min}\left(t\right)\leq \Delta U\left(t\right)\leq \Delta U_{max}\left(t\right),$$

where

$$\Delta U_{min}\left(t\right)=\left(\begin{array}{c} \Delta u_{min}\left(t|t\right)\\ \Delta u_{min}\left(t+1|t\right)\\ \vdots \\ \Delta u_{min}\left(t+N_{c}-1|t\right) \end{array}\right),\;\Delta U_{max}\left(t\right)=\left(\begin{array}{c} \Delta u_{max}\left(t|t\right)\\ \Delta u_{max}\left(t+1|t\right)\\ \vdots \\ \Delta u_{max}\left(t+N_{c}-1|t\right) \end{array}\right).$$

Accordingly, Equations (20), (25) and (26) yield the following quadratic programming problem

(27) $$\begin{array}{c} J\left(t\right)=\left(\begin{array}{cc} \Delta U^{T}\left(t\right) & \varepsilon \end{array}\right)H\left(t\right){\left(\begin{array}{cc} \Delta U^{T}\left(t\right) & \varepsilon \end{array}\right)}^{T}+F\left(t\right){\left(\begin{array}{cc} \Delta U^{T}\left(t\right) & \varepsilon \end{array}\right)}^{T},\\ s.t.\;U_{min}\left(t\right)\leq E\Delta U\left(t\right)+U\left(t-1\right)\leq U_{max}\left(t\right),\\ \Delta U_{min}\left(t\right)\leq \Delta U\left(t\right)\leq \Delta U_{max}\left(t\right). \end{array}$$

Sving Equation (27) in each control cycle leads to a series of control increments in the control time domain:

(28) $$\Delta U^{*}\left(t\right)=\left(\begin{array}{cccc} \Delta u^{*}\left(t|t\right) & \Delta u^{*}\left(t+1|t\right) & \cdots & \Delta u^{*}(t+N_{c} \end{array}-1)\right),$$

Furthermore, the first element in the sequence was adopted for the actual control increment

(29) $$u^{*}\left(t|t\right)=u\left(t-1|t\right)+\Delta u^{*}\left(t|t\right).$$

Finally, by repeating the above process in each control cycle, the desired trajectory is tracked.

### 3.3. Streering Strategy

**Figure 6:**
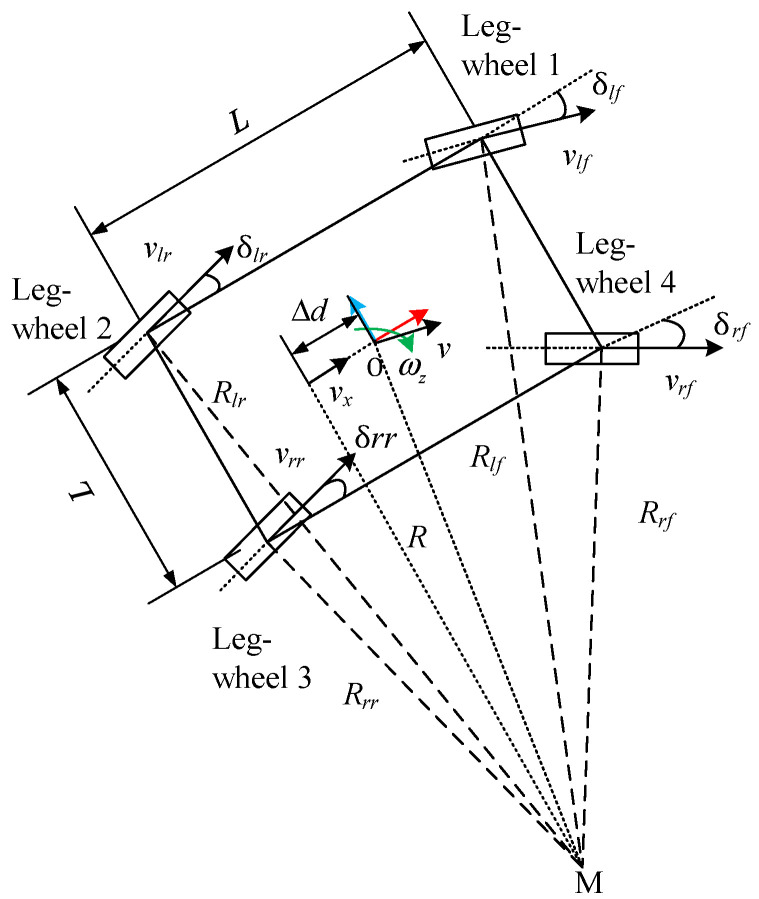
The steering strategy.

Using the aforementioned MPC method, we can obtain the optimal control inputs, $u^{*}\left(t|t\right)=\left(v_{x}^{B},\;\omega_{z}^{B}\right)$. Furthermore, the speed and the steering angle of each wheel need to be derived. In the present study, the steering strategy in which all of the wheels make the uniform circular motion was adopted, as seen in Figure 6.

The point M is the steering center and the steering radius of the robot *R* is

(30) $$R=\frac{v_{x}^{B}}{\;\omega_{z}^{B}},$$

where$\;\omega_{z}^{B}\neq 0$. Furthermore, the steering radii of the four wheels are written as

(31) $$\{\begin{array}{c} R_{rf}=\sqrt{{\left(\frac{L}{2}-\Delta d\right)}^{2}+{\left(R-\frac{L}{2}\right)}^{2}}\\ R_{lf}=\sqrt{{\left(\frac{L}{2}-\Delta d\right)}^{2}+{\left(R+\frac{L}{2}\right)}^{2}}\\ R_{rr}=\sqrt{{\left(\frac{L}{2}+\Delta d\right)}^{2}+{\left(R-\frac{L}{2}\right)}^{2}}\\ R_{lr}=\sqrt{{\left(\frac{L}{2}+\Delta d\right)}^{2}+{\left(R+\frac{L}{2}\right)}^{2}} \end{array}.$$

According to Ackermann’s principle, the wheel speeds and the steering angles are obtained as follows:

(32) $$\{\begin{array}{c} \omega_{rf}=\frac{\;\omega_{z}^{B}R_{rf}}{R_{w}}\\ \omega_{lf}=\frac{\;\omega_{z}^{B}R_{lf}}{R_{w}}\\ \omega_{rr}=\frac{\;\omega_{z}^{B}R_{rr}}{R_{w}}\\ \omega_{lr}=\frac{\;\omega_{z}^{B}R_{lr}}{R_{w}} \end{array}$$

(33) $$\{\begin{array}{c} \delta_{rf}=sign\left(k\right)\tan^{-1}\frac{\frac{L}{2}-\Delta d}{R-L/2}\\ \delta_{lf}=sign\left(k\right)\tan^{-1}\frac{\frac{L}{2}-\Delta d}{R+L/2}\\ \delta_{rr}=-sign\left(k\right)\tan^{-1}\frac{\frac{L}{2}+\Delta d}{R-L/2}\\ \delta_{lr}=-sign\left(k\right)\tan^{-1}\frac{\frac{L}{2}+\Delta d}{R+L/2} \end{array}$$

where $\omega_{rf}$, $\omega_{lf}$, $\omega_{rr}$, and $\omega_{lr}$ are the wheel speeds, $\mathrm{and}\;\delta_{rf}$, $\delta_{lf}$, $\delta_{rr}$, and $\delta_{lr}$ are the steering angles. Here, sign(k) is a signum function, and sign(k)=−1 when the wheel rotates clockwise; sign(k)=1 when the wheel rotates anticlockwise. When $\;\omega_{z}^{B}=0$ in Equation (30), the four steering angles are all zero, i.e., $\delta_{rf}=\delta_{lf}=\delta_{rr}=\delta_{lr}=0$.

### 3.4. Wheel Speed Allocation (WSA)

The WSA module calculates the suitable speed for each wheel according to the motion characteristics of the whole robot on rough terrain.

First, according to the kinematic equations, the linear velocity of the wheel center and the angular velocity of the lower leg (i.e., Frame 4) in Figure 4 are written as

(34) (viwωiw)=(RB4iRB4iSiw0RB4i)(vi0Bωi0B)+R04iJi(θ˙i1θ˙i2θ˙i3θ˙i4),

where $S_{iw}$ is the position vector of the wheel center with respect to the body frame; RB4i is the rotation transformation matrix from the body frame to frame 4; R04i is the rotation transformation matrix from the leg frame to frame 4; $J_{i}$ is the Jacobian matrix with respect to the leg frame; *i* = 1–4 denotes the leg number; and $v_{i0}^{B}$ and $\omega_{i0}^{B}$ are the linear and angular velocities of the leg frame with respect to the body frame, which are given by

(35) $$\left(\begin{array}{c} v_{i0}^{B}\\ \omega_{i0}^{B} \end{array}\right)=\left(\begin{array}{c} \omega^{B}\times \left(O_{0i}-M\right)\\ \omega^{B} \end{array}\right),$$

where $\omega^{B}$ is the angular velocity of the body.

Second, the ideal (no-slip) linear velocity of the wheel center comes from the driving motor and the rotation of the lower leg, which can be denoted by

(36) $$\left|\left|v_{iw}\right|\right|=\left(\omega_{iwz}+\omega_{id}\right)R_{w},$$

where $\omega_{iwz}$ is the projection of $\omega_{iw}$ on the direction of the wheel axis.

Accordingly, the rotational speeds of the wheel motors are obtained by

(37) $$\omega_{id}=\frac{\left|\left|v_{iw}\right|\right|}{R_{w}}-\omega_{iwz}.$$

## 4. Results and Discussion

### 4.1. Simulations

The numerical program of the control strategy was first developed using MATLAB software. Then, the joint simulation model was established by SIMULINK and UG Motion software. UG motion software provides the joint angles as well as the pitch and roll angles for each control block. In the meantime, the control blocks calculate the joint angles and wheel speeds and provide them to the virtual prototype. There are some system and control parameters that can be grouped into three categories, namely the input parameters, the output parameters, and the control parameters, as seen in Table 2. The input parameters include the points on the reference path, the desired yaw angle of the robot body, and the desired linear and angular velocities of the robot body. The output parameters are the linear and angular velocities of the robot body from the MPC and the steering angles and the wheel speeds. In addition, there are control parameters, including the prediction and control horizons, the weight coefficient and the relaxation factor, and the PID parameters to control the linear and angular velocities of the robot body.

**Figure 7:**
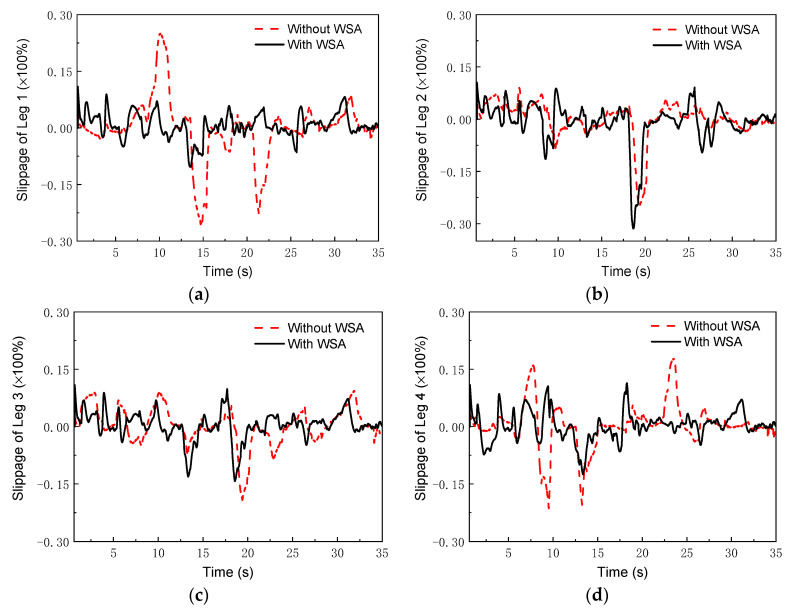
Slippage in simulations: (**a**) leg 1; (**b**) leg 2; (**c**) leg 3; (**d**) leg 4.

First, the WSA module was verified. For the simulation, the terrain included two trapezoid and two arc obstacles. The posture as well as the linear and angular velocities of the body, the joint angles and the angular velocities of the joints, and the driving speeds of the wheels were all measured using the virtual model in UG Motion software. Note that the terrain with the obstacles led to the changes of rover attitude angles (*α*, *β*). Here, the terrain-adaptive algorithm in [27] was adopted to control the rover attitude. With this algorithm, the robot attitude was almost kept unchanged in irregular terrains. Then, the practical linear velocity of the wheel centers and the practical angular velocity of the wheels were calculated according to Equations (36) and (37). Therefore, the slip percent could be obtained from

(38) $$\mu =\frac{\left(\left(\omega_{id}+\omega_{iwz}\right)R_{w}-v_{i}\right)}{\left(\omega_{id}+\omega_{iwz}\right)R_{w}}\times 100\%,$$

where *R_w_* is the radius of the wheel; $\omega_{id}$ is the practical angular velocity of the wheel, which can be measured by the wheels’ encoders; and $v_{i}$ is the practical linear velocity of the wheel center. Figure 7 shows the comparison of the slippage percentages with and without the WSA module. Without the WSA module, the slippage reached up to 0.25, while with the WSA, the maximum of the slippage was less than 0.13. It was found that wheel slip was obviously decreased by the WSA component.

**Figure 8:**
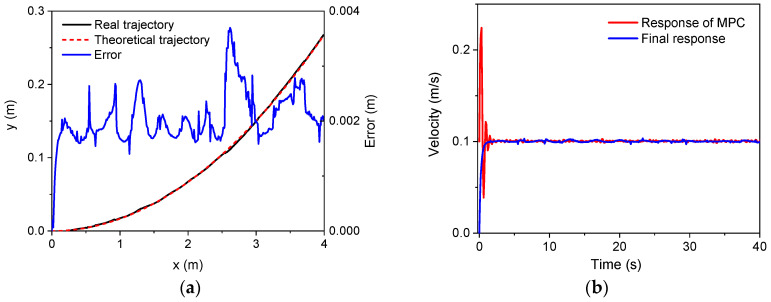
The simulation results (*v* = 0.1 m/s): (**a**) trajectory; (**b**) velocity.

**Figure 9:**
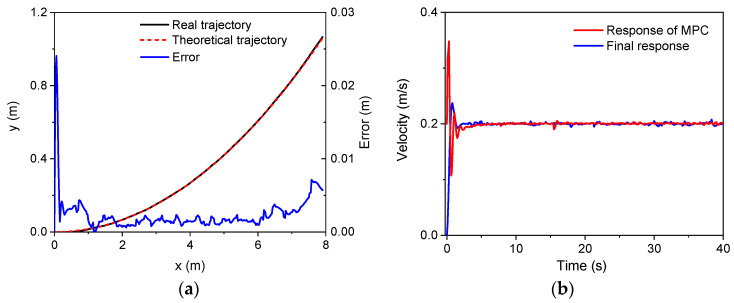
The simulation results (*v* = 0.2 m/s): (**a**) trajectory; (**b**) velocity.

**Figure 10:**
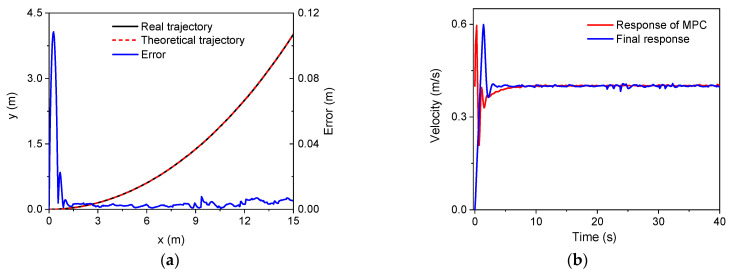
The simulation results (*v* = 0.4 m/s): (**a**) trajectory; (**b**) velocity.

Second, the trajectory tracking based on MPC was verified. An arc trajectory with a radius of 30 m in the plane was selected for the validation simulation. In the simulations, there were three speeds, i.e., 0.1 m/s, 0.2 m/s, and 0.4 m/s. The control parameters for the simulations of the linear trajectory were: *N_p_* = 6; *N_c_* = 3; ρ=10; ε=0 for the lower limit and ε=10 for the upper limit. The PID parameters for the linear velocity were set as: $k_{p1}=2,\;k_{i1}=1,\;k_{d1}=0$. Since there was only a linear velocity in the body frame, the PID module for the control of angular velocities did not work. Figure 8, Figure 9 and Figure 10 show the trajectory tracking results for three speeds. It was found that the robot could track the corresponding target values within a short period of time under the three different speeds. There was a large increase in trajectory errors at the beginning of the tracking process. The reason for this is that the initial direction of the target speed was the same as the *x* axis in the global coordinate system. There was an obvious delay before the actual speed reached the target value, and the speed error was relatively larger at the beginning. Furthermore, it was found that there were obvious overshoots in the velocity responses from the MPC method in the beginning. These overshoots facilitated trajectory tracking, and thus, the forward velocity of the robot could approximate the desired value quickly. In the meantime, the overshoot increased as the desired speed increases. The overshoot at 0.4 m/s was the largest one among the three forward speeds. It should also be noted that the final velocity response errors increased as the target speed increased. However, as a whole, the trajectory errors for the three speeds were all relatively small, validating the MPC module and the whole control strategy.

**Figure 11:**
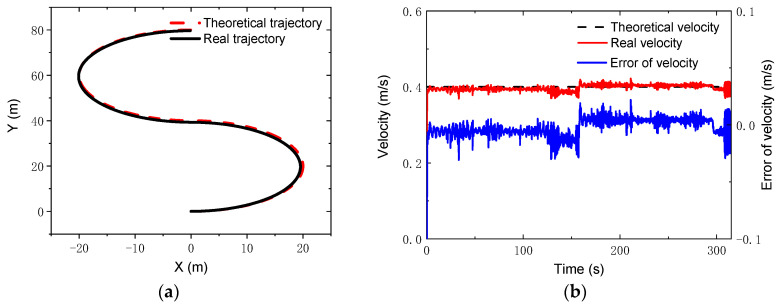
S-type trajectory and velocity: (**a**) trajectories; (**b**) velocities and errors.

**Figure 12:**
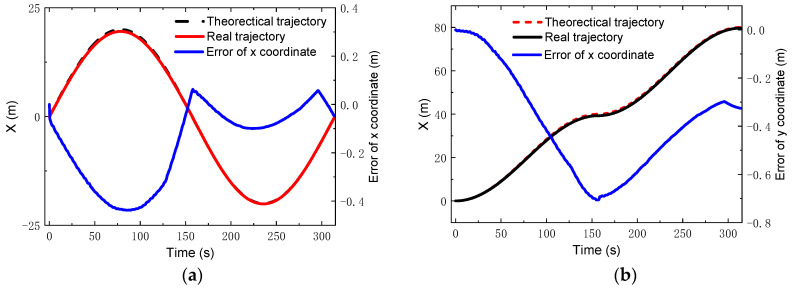
Coordinates and errors for S-type trajectory: (**a**) *x* coordinate; (**b**) *y* coordinate.

To further validate the control strategy, a more complicated trajectory, i.e., an S-type trajectory was selected for the tracking simulations. The S-type trajectory consisted of two semicircles with a radius of *R* = 20 m, which can be described as follows:

(39) $$x_{cr}^{w}=\{\begin{array}{l} R\sin\left(\omega_{z}t\right),\;0\leq t<T_{s}\\ R\sin\left[-\omega_{z}\left(t-T_{s}\right)\right],T_{s}\leq t\leq 2T_{s} \end{array},$$

(40) $$y_{cr}^{w}=\{\begin{array}{l} R-R\cos\left(\omega_{z}t\right),\;0\leq t<T_{s}\\ 2R-R\cos\left(\omega_{z}T_{s}\right)-R\cos\left[-\omega_{z}\left(t-T_{s}\right)\right],T_{s}\leq t\leq 2T_{s} \end{array},$$

(41) $$\gamma_{cr}^{w}=\{\begin{array}{l} \omega_{z}t,\;0\leq t<T_{s}\\ \omega_{z}T_{s}-\omega_{z}\left(t-T_{s}\right),T_{s}\leq t\leq 2T_{s} \end{array},$$

where $v_{x}=0.4\;m/s$; $\omega_{z}=v_{x}/R$; $T_{s}=\pi /\omega_{z}$. During the simulation, the control parameters were set as: *N_p_* = 6; *N_c_* = 3; ρ=10; ε=0 for the lower limit and ε=10 for the upper limit. The PID parameters for the linear velocity were set as: $k_{p1}=0.7,\;k_{i1}=0,\;k_{d1}=5$. Figure 11 gives the comparisons of the theoretical and real trajectories and velocities. It was found that the robot could track well the reference trajectory and the reference velocity when running along the S-type trajectory. Furthermore, the errors in the x and y coordinates were very small, the relative error of which were less than 2% and 1.75%, respectively, as seen in Figure 12.

**Figure 13:**
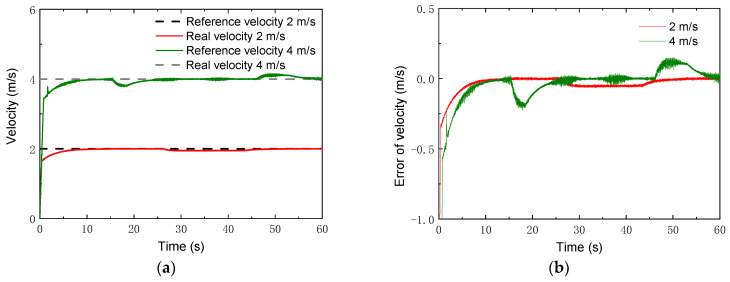
Velocity results with higher speeds: (**a**) velocity; (**b**) error of velocity.

**Figure 14:**
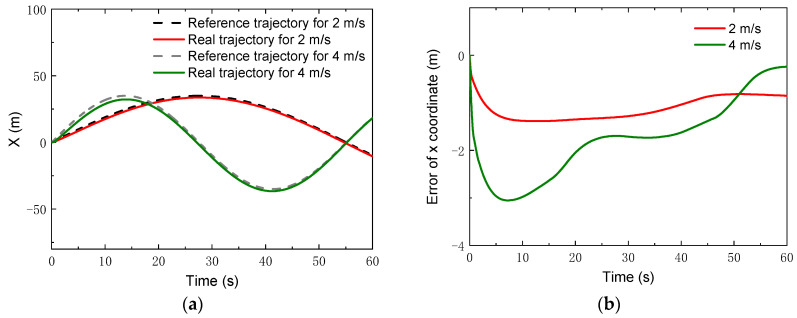
*x* Coordinates with higher speeds: (**a**) *x* coordinate; (**b**) error of *x* coordinate.

**Figure 15:**
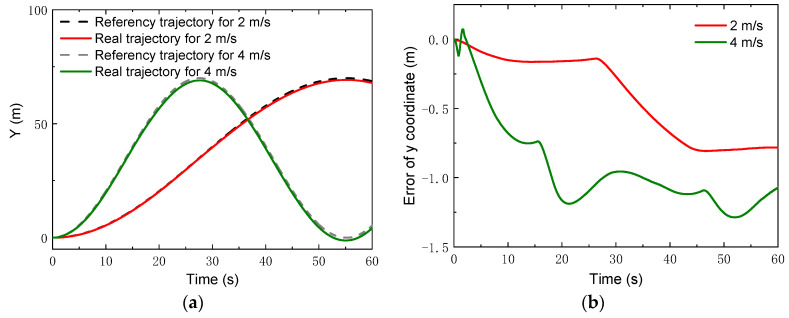
*y* Coordinates with higher speeds: (**a**) *y* coordinate; (**b**) error of *y* coordinate.

In addition, tracking simulations for high speeds were also carried out. Two speeds, i.e., $v_{x}=2\;m/s$ and 4 m/s, were chosen, which were ten times as large as the speeds in the previous simulations. A circle trajectory with a radius of *R* = 35 m was selected for the simulations, which can be described by

(42) $$\{\begin{array}{l} x_{cr}^{w}=R\sin\left(\omega_{z}t\right)\\ y_{cr}^{w}=R-R\cos\left(\omega_{z}t\right)\\ \gamma_{r}^{w}=\omega_{z}t \end{array},$$

where $\omega_{z}=v_{x}/R$. The control parameters for the simulations of the linear trajectory were set as: *N_p_* = 6; *N_c_* = 3; ρ=10; for the lower limit and ε=10 for the upper limit. The PID parameters for the linear velocity were set as: $k_{p1}=2,\;k_{i1}=1,\;k_{d1}=0$. Figure 13 gives the changes of the real velocities. It was found that the robot could still track the reference velocity after a relatively short time. Moreover, with the control, the robot could track the reference trajectories of both the *x* coordinate and the *y* coordinate, depicted in Figure 14 and Figure 15. Compared to the lower speed, the position errors increased. However, the relative errors of the position points were small. The maxima of the relative position errors at 2 m/s and 4 m/s were less than 3% and 8.5%, respectively.

### 4.2. Experiments

**Figure 16:**
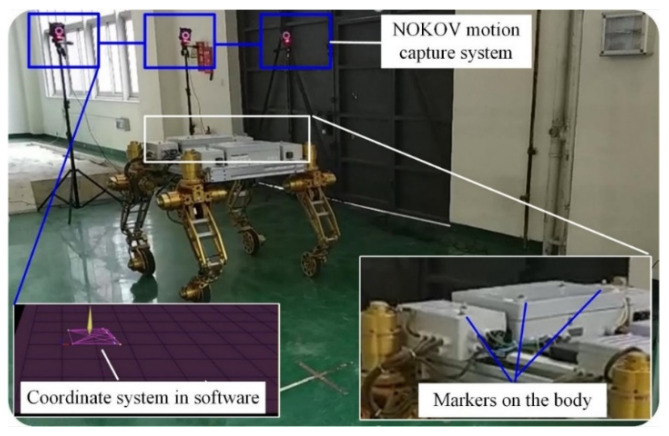
The experimental setup.

**Figure 17:**
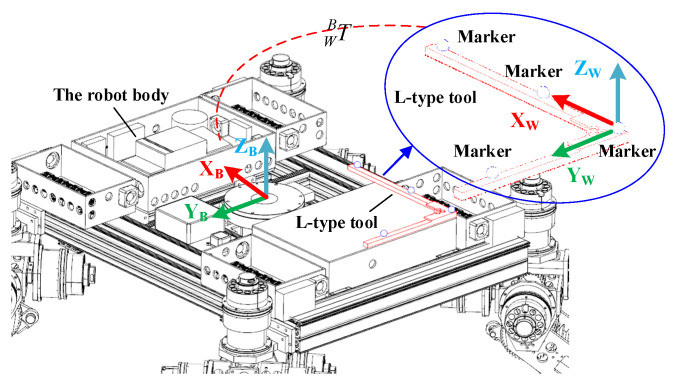
The calibration of the vision frame.

**Figure 18:**
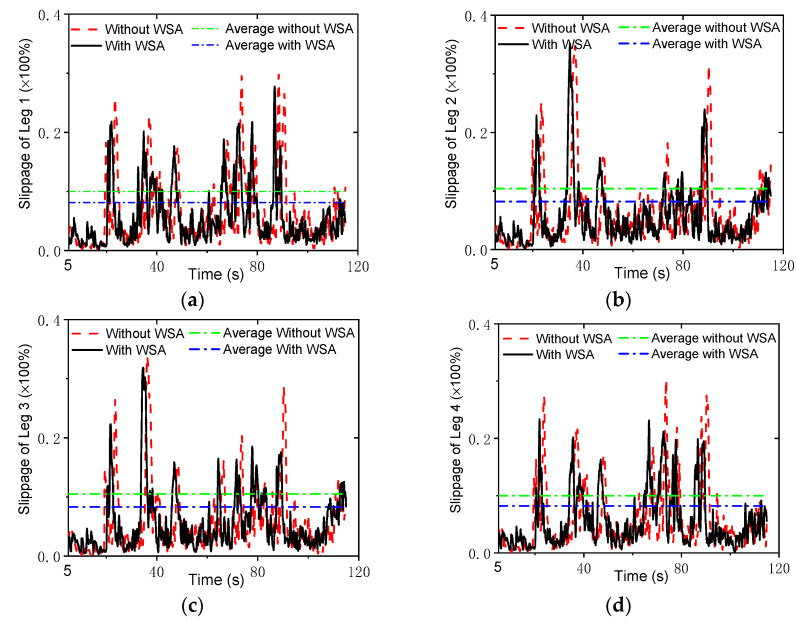
Slippage in the experiments: (**a**) Leg 1; (**b**) Leg 2; (**c**) Leg 3; (**d**) Leg 4.

**Figure 19:**
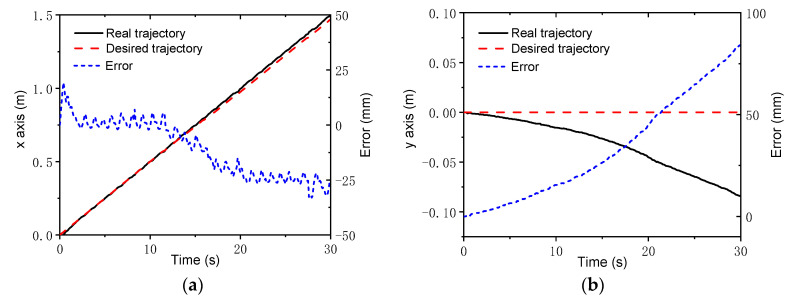
Experimental trajectory tracking results: (**a**) *x* axis; (**b**) *y* axis.

To further verify the control strategy, an experimental setup based on the NOKOV vision motion capture system was established that consisted of six cameras, as shown in Figure 16. An L-type tool was used for the benchmark calibration of the vision system. After there were enough cameras placed around the robot, the c vision capture system was calibrated. The L-type tool was mounted on the body, as seen in Figure 17. There are four markers on the L-type tool. The cameras recognize the markers, and thus, the vision frame (i.e., the global coordinate system) can be established. According to the geometrical relationship between the mounting location of the L-type tool and the robot’s body frame, the initial transformation matrix between the vision frame and the body frame could be obtained. Serval markers were bonded to the body of the rover, and thus, the body coordinate system could be established in the world coordinate system. Therefore, the real motion trajectory of the rover could be measured in real time and sent to the rover control system. To clarify the effectiveness of the WSA module, a terrain with a flat surface and two trapezoids was employed in the experiments. Since the terrain included obstacles, the terrain-adaptive algorithm [27] was adopted, similar to the simulations. During the experiments, the control parameters for the MPC module were set as: *N_p_* = 6; *N_c_* = 3; ρ=10; ε=0 for the lower limit and ε=10 for the upper limit. The PID parameters for the control of linear velocities were set as: $k_{p1}=8,\;k_{i1}=0,\;k_{d1}=0.2$. Figure 18 shows the slippage in the experiments. Note that the slippages of Leg 1 and 4 (Leg 2 and 3) are almost the same because they suffer the same terrain condition. It was found that the average slippage of all of the legs demonstrated an obvious decrease after WSA control, up to 20%. Figure 19 shows the experimental results of trajectory tracking. As it can be seen, the rover could strictly track the reference trajectory. The deflection with respect to the reference trajectory was less than 2% F.S., which is a relatively small error. Accordingly, the control strategy was validated by the experiments.

## 5. Conclusions

In this paper, a novel wheeled–legged planetary rover with four legs was proposed, and each leg had four DOFs with an actuated wheel. The articulated legs utilized a serial–parallel hybrid configuration, and it had the merits of both serial and parallel mechanisms. Moreover, the legs had a rigid–flexible coupling structure that could conform to unstructured terrain using both active and passive compliance. The kinematics equations of the rover were derived. Then, a control scheme including trajectory tracking, the steering strategy, and the WSA module was proposed. A trajectory tracking model based on MPC that could handle the line and arc trajectory with quite a good accuracy was established. In addition, the WSA was introduced into the control strategy to decrease the slippage. After that, to validate the control method, three groups of cosimulations, i.e., tracking an arc trajectory, tracking a S-type trajectory, and trajectory tracking with high speeds were carried out. Finally, trajectory tracking experiments were conducted through a vision motion capture system. It was found that the average slippage of all of the legs decreased obviously after WSA control, with a slippage up to 20% in our experiments. Moreover, the rover could strictly track the reference trajectory. With respect to the reference trajectory, the deflection was found to be less than 2% F.S., which is a relatively small error. Accordingly, the proposed control strategy was thoroughly verified by the simulations and experiments.

## Figures and Tables

**Table 1:** The D-H parameters.

	*θ* *_i_*	*d_i_*	α*_i_*	*a_i_*
1	*θ*_1_	0	*π*/2	0
2	*θ*_2_	0	−*π*/2	*L*_1_ = 87.5
3	*θ*_3_	0	0	*L*_2_ = 350
4	*θ*_4_	0	0	*L*_3_ = 310

**Table 2:** The system and control parameters.

Categories	Terminology	Definition
Input parameters	$x_{cr}^{w}$ $,\;y_{cr}^{w}$	Points on reference path
	$\gamma_{r}^{w}$	Desired yaw angle of body
	$v_{xr}^{B}$ $,\omega_{zr}^{B}$	Desired linear and angular velocities of body
Control parameters	*N_p_*, *N_c_*	Prediction and control horizons
	ρ, ε	Weight coefficient and the relaxation factor
	*k_p_*_1_, *k_i_*_1_, *k_d_*_1_	PID parameters for control of the linear velocity of body
	*k_p_*_2_, *k_i_*_2_, *k_d_*_2_	PID parameters for control of the angular velocity of body
Output parameters	$u^{*}\left(t|t\right)=\left(v_{x}^{B},\omega_{z}^{B}\right)$	The linear and angular velocities of the body
	$\delta_{rf}$ $,\delta_{lf}$ $,,\delta_{lr}$	Steering angles
	$\omega_{id}$.	Rotational speeds of the wheel motors

## Data Availability

Data will be made available upon reasonable request by the corresponding author.

## References

[1] Rankin A., Maimone M., Biesiadecki J., Patel N., Levine D., Toupet O. Driving curiosity: Mars rover mobility trends during the first seven years. Proceedings of the IEEE Aerospace Conference.

[2] Dodge R., Parsons D., Abid M., Chrystal K., Kartolov B. Dynamics associated with the Corer on M2020 Perseverance Rover. Proceedings of the IEEE Aerospace Conference.

[3] Zheng J., Gao H., Yuan B., Liu Z., Yu H., Ding L., Deng Z. (2018). Design and terramechanics analysis of a Mars rover utilizing active suspension. Mech. Mach. Theory.

[4] Michaud F., Letourneau D., Arsenault M., Bergeron Y., Cadrin R., Gagnon F., Legault M.A., Millette M., Paré J.F., Tremblay M.C. (2005). Multi-modal locomotion robotic platform using leg-track-wheel articulations. Auton. Robots.

[5] Hauser K., Bretl T., Latombe J.C., Harada K., Wilcox B. (2008). Motion Planning for Legged Robots on Varied Terrain. Int. J. Robot. Res..

[6] Grand C., Benamar F., Plumet F., Bidaud P. (2004). Stability and traction optimization of a reconfigurable wheel-legged robot. Int. J. Robot. Res..

[7] Smith J.A., Poulakakis I., Trentini M., Sharf I. (2010). Bounding with active wheels and liftoff angle velocity adjustment. Int. J. Robot. Res..

[8] Xu K., Wang S., Yue B., Wang J., Peng H., Liu D., Chen Z., Shi M. (2020). Adaptive impedance control with variable target stiffness for wheel-legged robot on complex unknown terrain. Mechatronics.

[9] He J., Gao F. (2015). Type Synthesis for bionic quadruped walking robots. J. Biol. Eng..

[10] He J., Gao F. (2020). Mechanism, actuation, perception, and control of highly dynamic multi-legged robots: A Review. Chin. J. Mech. Eng..

[11] Lewinger W.A., Harley C.M., Ritzmann R.E., Branicky M.S., Quinn R.D. Insect-like antennal sensing for climbing and tunneling behavior in a biologically-inspired mobile robot. Proceedings of the IEEE International Conference on Robotics and Automation.

[12] Daltorio K.A., Wei T.E., Gorb S.N., Ritzmann R.E., Quinn R.D. Passive foot design and contact area analysis for climbing mini-whegs. Proceedings of the IEEE International Conference on Robotics and Automation.

[13] Chen S.C., Huang K.J., Chen W.H., Shen S.Y., Li C.H., Lin P.C. (2014). Quattroped: A leg–wheel transformable robot. IEEE/ASME Trans. Mechantron..

[14] Kim Y.S., Jung G.P., Kim H., Cho K.J., Chu C.N. (2014). Wheel transformer: A wheel-leg hybrid robot with passive transformable wheels. IEEE Trans. Robot..

[15] Chen W.H., Lin H.S., Lin Y.M., Lin P.C. (2017). TurboQuad: A novel leg–wheel transformable robot with smooth and fast behavioral transitions. IEEE Trans. Robot..

[16] Kim Y., Lee Y., Lee S., Kim J., Kim H.S., Seo T. (2020). STEP: A new mobile platform with 2-DOF transformable wheels for service robots. IEEE/ASME Trans. Mechantron..

[17] Sun T., Xiang X., Su W., Wu H., Song Y. (2017). A transformable wheel-legged mobile robot: Design, analysis and experiment. Robot. Auton. Syst..

[18] Grotzinger J.P., Crisp J., Vasavada A.R., Anderson R.C., Baker C.J., Barry R., Blake D.F., Conrad P., Edgett K.S., Ferdowski B. (2012). Mars science laboratory mission and science investigation. Space Sci. Rev..

[19] Cordes F., Kirchner F., Babu A. (2018). Design and field testing of a rover with an actively articulated suspension system in a Mars analog terrain. J. Field Robot..

[20] Lamon P. (2008). 3D-position tracking and control for all-terrain robots. Adv. Robot..

[21] Chwa D. (2016). Robust distance-based tracking control of wheeled mobile robots using vision sensors in the presence of kinematic disturbances. IEEE Trans. Ind. Electron..

[22] Liang Z., Chen J., Wang Y. (2019). Equivalent acceleration imitation for single wheel of manned lunar rover by varying torque on earth. IEEE/ASME Trans. Mechatron..

[23] Krid M., Amar F.B. A dynamic based path tracking controller for a fast rover with independent steering and drive. Proceedings of the CLAWAR 2011.

[24] Yang H., Zhao H., Xia Y., Zhang J. (2021). Nonlinear MPC with time-varying terminal cost for tracking unreachable periodic references. Automatica.

[25] Liu X., Wang W., Li X., Liu F., He Z., Yao Y., Ruan H., Zhang T. (2022). MPC-based high-speed trajectory tracking for 4WIS robot. ISA Trans..

[26] Ding T., Zhang Y., Ma G., Cao Z., Zhao X., Tao B. (2022). Trajectory tracking of redundantly actuated mobile robot by MPC velocity control under steering strategy constraint. Mechatronics.

[27] Sun Y.L., He J., Xing Y. (2021). Multi-target coordinated control of wheel-legged Mars rover. Acta Aeronutica Astronaut. Sin..

[28] Li W., Ding L., Gao H., Tavakoli M. (2020). Haptic tele-driving of wheeled mobile robots under nonideal wheel rolling, kinematic control and communication time delay. IEEE Trans. Syst. Man Cybern. Syst..

[29] Chen C., Shu M., Wang Y., Ding L., Gao H., Liu H., Zhou S. (2021). Simultaneous control of trajectory tracking and coordinated allocation of rocker-bogie planetary rovers. Mech. Syst. Signal Proc..

